# Recognition of depression in children in general hospital-based paediatric units in Kenya: practice and policy implications

**DOI:** 10.1186/1744-859X-8-25

**Published:** 2009-10-28

**Authors:** David M Ndetei, Lincoln I Khasakhala, Victoria N Mutiso, Anne W Mbwayo

**Affiliations:** 1 Africa Mental Health Foundation (AMHF), Nairobi, Kenya

## Abstract

**Background:**

Physical disorders are commonly comorbid with depression in children attending general medical facilities. However, the depression component is rarely recognised.

**Methods:**

A questionnaire on sociodemographics and history of presenting medical conditions was administered together with the Children's Depression Inventory (CDI) to all 11-year-old to 17-year-old children attending at nine medical facilities.

**Results:**

In all, 408 children were recruited from 9 health facilities. Whereas the clinicians diagnosed a mental disorder in only 2.5% of the sample studied, 41.3% had CDI scores that suggested mild to moderate depression. The highest proportion of children with depressive symptomatology was found at the Kenyatta National and Teaching Referral Hospital.

**Conclusion:**

Although prevalence rate for depression among children is high, detection rates remain low. This finding has clinical practice and policy implications within and outside Kenya.

## Background

Comorbid mental and physical disorders have been previously reported in children [1]. Early diagnosis is particularly critical [2] since the onset of most mental illnesses occurs in adolescence or early adulthood [3].

There is an association between chronic illnesses and paediatric psychiatric disorders [4-6] and in particular depressive disorders [6]. In Africa in general and Kenya in particular, there is no published data on the comorbidity of physical illnesses and depression in paediatric populations in general medical facilities. This study aims to fill that gap.

## Methods

**Figure 1:**
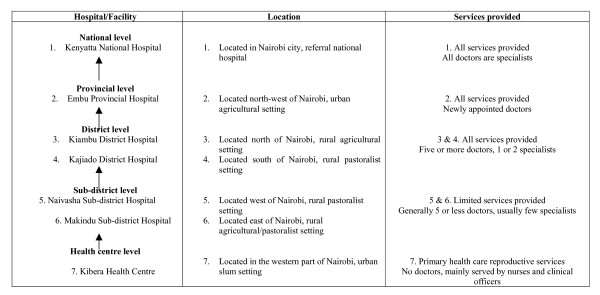
**The referral structure of public medical facilities in Kenya**. Two private health facilities were also included in the study. Magadi hospital is located in a rural pastoralist setting, north of Nairobi, and Kikuyu hospital located west of Nairobi is found in a predominantly agricultural rural setting. Both are served by privately employed doctors and provide elementary health services.

A descriptive cross-sectional survey was conducted in a purposive stratified sample of nine health facilities representing a spectrum of considerations, including different economic environments within which the facilities were located (industrial, agricultural, nomadic, rural and urban), as well as the different training levels of medical personnel. To represent the above considerations, the geographical locations of the health facilities were selected on the basis of their convenient proximity and therefore accessibility (given the limited resources for this study) and were all within a 200 km radius to Nairobi, the capital city of Kenya. The different public health care levels (systems) in Kenya and a brief description of the facilities studied have been described previously [7] (figure 1).

All the facilities, except the health centres, offer both inpatient and outpatient services. Attendance at these facilities depends on their locality and is determined by accessibility, affordability and availability of services required. All those attending the national hospital are usually referrals that cannot be managed in the other facilities. Also included in the study were one faith-based, not for profit hospital (Kikuyu) and one private institutional hospital (Magadi). Kikuyu Hospital, located in a semirural setting about 10 km from Nairobi, is a national centre for medical and surgical problems of the eye. The Magadi Hospital, located about 90 km to the south of Nairobi in the rural interland of the Masai community, treats (for free) staff and their relatives of a multinational mining company located there. The staff are drawn from across the country. It also provides free treatment to the surrounding nomadic Masai community as part of its social responsibility programme. It is better resourced than most public facilities with the exception of Kenyatta National Hospital (KNH). All the above facilities, except the Magadi Hospital, raise minimal but highly subsidised fees for their services.

Ethical clearance for the study was obtained from the Kenyatta National Hospital Sciences and Ethics Review Committee.

The data was collected over a 4-week period in November 2005. During this period, 490 children (excluding those in psychiatric wards or clinics) attended the selected health facilities as inpatients or outpatients. The age range of the children targeted for inclusion in this study was 11 to 17 years, this being within the age range for the instrument used for assessing depression [8] and also given that those 18 and above would normally not be seen in the paediatric facilities. For the children who were not too ill to participate, informed consent was obtained from parents or guardians. Once the parents/guardians signed the consent form, the children were then requested for their informed assent to participate in the study.

A questionnaire on the children's sociodemographic characteristics was used to elicit information on age, gender, religious affiliation, level of education and type and duration of current medical problem(s). Such information is routinely sought in clinical settings and is readily provided by respondents. This questionnaire was administered together with the Children's Depression Inventory (CDI) [8]. The instrument was administered by fourth and fifth year medical students and registered nurses, who underwent a 1 day training from the lead author of this paper who was also the principal investigator.

The CDI is a 27-item self-report instrument designed to assess cognitive, behaviour and neurovegetative signs of depression in children. Each item consists of three statements from which the child is instructed to choose the one statement that best describes them over the previous 2 weeks. Each question is designed to assess specific symptoms of depression and the three choices range from mild or limited symptomatology to severe or maladaptive symptomatology. Each item is scored 0, 1 or 2, with a score of 2 representing the most severe choice. The total scores on the CDI ranges from 0 to 54. To overcome problems that may arise because of a child or adolescent having a poor reading level, the research assistant read the items to every participating child or adolescent. The statements in the CDI were read up to a maximum of three readings if the child did not understand the questions. A score of non-response was entered when the respondent was unable to provide appropriate responses because of language difficulties after the third reading.

The participants were also asked what they thought they were suffering from. They were also asked whether the doctor had given them the opportunity to talk about their diagnosis and, if so, what the doctor had told them their diagnosis was. The final clinical diagnoses were extracted from the clinical notes.

Double data entry and cleaning were performed and analysis performed using SPSS v. 16 (SPSS, Chicago, IL, USA).

## Results

A total of 408 out of 490 (83.3%) children from 9 facilities, all being managed for physical disorder(s), met the inclusion criteria. Exclusions were made on the basis of being too ill to participate. No consent or assent was denied. The mean age of the sample was 15.54 years (range 11 to 18 years (see Discussion for information on the unintended inclusion of some 18 year olds)). There were more boys (55.4%) than girls, and majority (90.7%) of the sample were Christians. In all, 156 (38.2%) of the subjects had attained primary school level education (1 to 8 years of formal education) while 57.1% had a secondary school level education (1 to 4 years post primary). These results are summarised in Table 1. From the clinical notes, duration of the various physical illnesses varied greatly (Table 2) and included the whole spectrum of the various body systems and causes. The longest durations were in patients at the KNH and Kikuyu hospital (both of which are specialised referral hospitals). Physical injuries were listed amongst the physical illnesses.

**Table 1:** Sociodemographic characteristics of children in general medical facilities* (%)

**Variables**	**All sites (n)**	**KNH (n)**	**Embu (n)**	**Kiambu (n)**	**Kikuyu (n)**	**Kajiado (n)**	**Kibera (n)**	**Makindu (n)**	**Naivasha (n)**	**Magadi (n)**
Age in years	408	117	27	134	24	33	23	28	12	10
11 to 13	13.7	12.9	11.5	10.3	21.7	21.3	21.7	18.5	28.5	20.0
14 to 16	56.4	58.1	57.7	57.4	56.4	63.6	56.4	40.7	28.5	40.0
17 to 18	29.9	29.1	30.7	32.4	21.7	15.1	21.7	40.7	35.7	40.0
Gender	383	111	23	128	22	31	22	24	12	10
Male	59.1	62.5	52.2	70.8	54.5	45.2	54.5	52.2	42.9	0
Female	40.9	37.5	47.8	29.2	45.5	54.8	45.5	47.8	57.1	100
Religion	396	111	25	130	24	31	22	27	11	10
Christian	93.5	95.7	96	90.9	100	87.4	100	100	69.2	100
Muslim	5.5	3.4	4	9.1	0	12.6	0	0	7.7	0
Others	1.2	0.9	0	0	0	0	0	0	23.1	0
Education level^a^	395	115	27	128	24	30	22	27	12	10
College	1.3	0	0	3.1	0	0	0	3.8	0	0
Primary	39.6	49.6	66.7	18.5	45.5	40	45.5	53.9	85.7	60
Secondary	59.1	50.4	33.3	78.4	54.5	60	54.5	42.3	14.3	40

^a^Primary level = 1 to 8 years of formal education; secondary level = 1 to 4 years of post-primary education; college level = post-secondary education.

KNH = Kenyatta National Hospital.

*from Figure 1, these different facilities represent different shades of characteristics

**Table 2:** Duration of illness for which the child was seeking medical services (%)

**Duration of illness**	**All sites (n = 305)**	**KNH^a ^(n = 110)**	**Embu (n = 24)**	**Kiambu (n = 63)**	**Kikuyu^a ^(n = 14)**	**Kajiado (n = 25)**	**Kibera (n = 23)**	**Makindu (n = 25)**	**Naivasha (n = 11)**	**Magadi (n = 10)**
1 to 6 days	30.2	13.6	20.8	50.8	28.6	24.0	60.9	36.0	27.3	40.0
1 to 3 weeks	23.9	19.1	58.3	31.7	14.3	20.0	13.0	12.0	18.2	30.0
1 to 3 months	15.7	17.3	12.5	9.5	14.3	24.0	17.4	24.0	18.2	0
4 to 6 months	4.9	8.2	0	1.6	0	8.0	0	4.0	18.2	0
7 to 9 months	3.0	4.5	0	3.2	0	4.0	0	4.0	0	0
10 to 11 months	0.7	0	4.2	0	0	0	0	0	0	10
≥ 1 year	21.6	37.3	4.2	1.6	42.8	20.1	8.7	20.0	18.2	20.0

^a^KNH takes referrals for complicated cases from across the nation, while Kikuyu also has referrals for eye problems from across the country.

KNH = Kenyatta National Hospital.

A total of 367 (90.0%) subjects responded to all the questions asking about their clinical diagnoses, of whom 336 (82.4% of total sample) thought they knew what they were suffering from even though the clinicians had disclosed the diagnoses to only 58.1% (n = 237) of them. However, there was a wide variation across the nine sites. In all, 164 subjects (40.2%) had been given a chance to ask questions about their conditions. At the time of the interview 181/408 (44%) of the children had been told by their doctors what they were suffering from. The working clinical diagnosis had been entered in the clinical rules in 331/408 (81%) of the cases.

'Depression' (two children), 'mental problems' (one child), 'insomnia' (three children) and epilepsy (one child) were the only self-reported mental problems, reported by only seven children. According to the case notes, only 10 children were diagnosed with conditions related to mental health, which included depression (4 cases), epilepsy (2 cases), and 1 case each of panic attack, 'psychosis', schizophrenia and stress; this represented a clinician detection rate of 2.5% for the whole spectrum of mental disorders, and specifically for depression only 1% (n = 4). Of these 10 cases, 4 were at the KNH and had been referred to liaison psychiatry while the other 3 were from one district hospital and had also been referred for psychiatric consultation. The remaining 3 were in other district hospitals and had not been referred for psychiatric consultation. Those referred for psychiatric consultation had not yet been seen by a psychiatrist and were still at the general facilities.

A total of 344 (84.3%) subjects responded appropriately to all the items of the CDI (Table 3). More than half (n = 202; 58.7%) had normal scores on the CDI while the remainder (41.3%) scored positively for depressive symptoms, suggesting that they suffered from some degree of depression. Scores for severe depression were not recorded at any of the sites, although subjects from five facilities scored for moderate depression on the CDI. Depression was more but not significantly associated with long-term illnesses. All the children receiving a diagnosis of depression by the clinicians also scored positive for depression on CDI.

**Table 3:** Depressive symptoms in children (%)

	**All sites**	**KNH**	**Embu**	**Kiambu**	**Kikuyu**	**Kajiado**	**Kibera**	**Makindu**	**Naivasha**	**Magadi**
Patients, n	344	103	23	112	23	19	21	24	9	10
CDI scores:										
Normal, <10	58.7	49.5	30.4	60.7	78.3	63.2	95.2	70.8	33.3	60.0
Mild, 11 to 26	36.0	47.6	56.5	32.1	21.7	21.1	4.8	29.2	55.6	40.0
Moderate, 27 to 40	5.2	2.9	13.0	7.1	0	15.8	0	0	11.1	0

CDI = Children's Depression Inventory; KNH = Kenyatta National Hospital.

## Discussion and Conclusion

This study has limitations, the most important being that the geographical area of study was selected for convenience, although efforts were made to pick a stratified sample within the area of the study. However, given the proximity to the capital where most of the resources are located, the findings of this study can be safely assumed to reflect the best-resourced area in the country and, therefore, the situation for the rest of the country could presumably only be worse. The other limitation is that this is not an epidemiological or prevalence study of paediatric mental disorders but an attempt to gauge sensitivity to paediatric mental disorders and in particular paediatric depression on the part of the clinicians in the general facilities. Therefore, the issue of cultural variations in the diagnosis of paediatric depression is not critical to this study. As is the case with most psychometric instruments, the psychometric properties of the CDI in the context of Kenya and most African countries have not been documented; but again this was not an epidemiological study for purposes of crosscultural comparisons.

Further mitigation against these caveats is the evidence suggesting that there are more similarities than differences between Kenya and European countries in the epidemiological patterns of paediatric mental disorders [9] and in psychometric properties of commonly used instruments [10].

With above caveats in mind, the results can be discussed.

The unintended inclusion of a few children aged 18 years in the study was an artefact of the thin transition from childhood to adulthood, and therefore some 18-year-olds were being treated in paediatric setups.

More boys than girls participated in the study, and this could possibly be explained by the fact that one of the most common reasons for seeking health services in this study was orthopaedic/soft tissue injuries, which are more common in boys than in girls within this age band. As has been observed by another study, boys are more likely to experience most kinds of injuries and to be involved in behaviours that are highly correlated with injury [11]. However, this finding could also suggest that girls are highly discriminated against in access to hospital treatment, as one study in India showed [12]. The higher proportion of girls than boys in some of the facilities located in drier areas was not surprising as the populations in these areas are mainly nomadic pastoralists. Among these communities it is the boys who are traditionally assigned the roles of looking after livestock. At the time of the survey, there was a nationwide drought, which necessitated movement of livestock to pastures that were further away.

The proportion of Muslims in the study was lower than that found in the general population, where Muslims account for about 10% of the Kenyan population, as reported in the last census [13] and 6 years prior to the data collection, suggesting that the sample may not have been representative. There were more children with secondary school education than those with primary school education. This could be explained by the fact that in Kenya, the 8-year primary schooling period begins at the age of 6 years and ends at 14 years. The number of children in primary school was therefore proportionate to the number of children aged 14 years and below.

Clinicians detected only a small proportion of children with mental disorders in general (2.5%) and 1% for depression compared to the 41.3% depression detected on the CDI. These paediatric findings are similar to the 4.1% clinician pick-up rate for mental disorders in general and 42% instrument assisted pick-up rate, respectively, found in the adult population in the same facilities using different instruments appropriate for depression in adults [7]. This finding suggests that paediatric mental disorders and depression in particular largely go unrecognised, as has been reported elsewhere [2,3]. The highest prevalence rates of mild to moderate depression according to the CDI were reported at the national referral hospital, and since it is to such a facility that chronic cases are referred to, it could be that the children with chronic illnesses also suffered higher levels of depression; also similarly reported elsewhere [4-6]. This is despite the fact that the clinical pick-up rate at the Kenyatta National Hospital for mental disorders in general was 4/103 (3.9%) in a facility with the highest concentration of the most specialised doctors in the country, which also happens to be the leading teaching hospital in the country. The ripple effects are obvious: it produces personnel for the rest of the country who hardly recognise paediatric mental disorders in general medical facilities.

These findings call for appropriate practice and policy measures to increase the awareness, recognition and management of paediatric mental disorders by clinicians, patients and their parents/guardians. Given the dearth of psychiatrists in most countries in Africa, Kenya included [14], there is an urgent need to adapt instrument-assisted screening and diagnosis. This calls for validation of various internationally recognised instruments (for purposes of international comparisons). Although there has been an attempt in Kenya in that direction [10], more needs to be done. There is also the need for continuing medical education (CME) to sensitise all stakeholders in paediatric mental disorders in community and all clinical settings. All of these factors have profound preventive significance given that most adult disorders start in childhood, adolescence and early adulthood [2,3]. Public awareness campaigns in schools for both teachers and students are necessary.

The findings of this study have relevance to most of Africa and also concern global inequities in health services. Most countries in sub-Saharan Africa and outside South Africa and Mauritius, Kenya included, are similar socioeconomically, demographically (pyramid-shaped population structure) and culturally, but relatively worse off than Kenya in terms of human resources in mental health [14].

It can therefore be speculated that, as in Kenya, children in most African countries who are seen in general medical facilities are not routinely screened for mental health disorders and therefore these conditions go undiagnosed and unmanaged. On a global scale, the findings of this study not only contribute to the available data but also draw attention to global health inequities and in this particular case, inequalities regarding mental health services for children despite evidence for similar mental disorder epidemiological patterns in children [9].

## Competing interests

The authors declare that they have no competing interests.

## Authors' contributions

DMN contributed to the conception and design of the study, was involved in drafting the manuscript and revising it critically for intellectual content and was also involved in training the data collectors. LIK participated in acquisition, analysis and interpretation of data and was involved in drafting the manuscript and revising it critically for intellectual content. VNM participated in acquisition, analysis and interpretation of data and was involved in drafting the manuscript. AWM participated in drafting and editing the manuscript.
